# Mesenchymal Stem Cells Provide Neuroprotection by Regulating Heat Stroke-Induced Brain Inflammation

**DOI:** 10.3389/fneur.2020.00372

**Published:** 2020-05-05

**Authors:** Yu Zhang, Zihui Deng, Yun Li, Rui Yuan, Mengmeng Yang, Yan Zhao, Lu Wang, Feihu Zhou, Hongjun Kang

**Affiliations:** ^1^Department of Critical Care Medicine, General Hospital of the Chinese People's Liberation Army, Beijing, China; ^2^Biochemistry Department of Graduate School, General Hospital of the Chinese People's Liberation Army, Beijing, China

**Keywords:** heat stroke, mesenchymal stem cell, neuroinflammation, astrocyte, inflammatory reactions

## Abstract

Heat stroke (HS) is the most acute type of heat illness accompanied with serious central nervous system (CNS) dysfunction. Despite the pathological process being clearly studied, effective treatment is deficient. Currently, mesenchymal stem cells (MSCs) have been demonstrated to have neuroprotective effects as there are no old ones. Thus, the purpose of the present study was to explore the neuroprotective effects and mechanisms of MSCs against HS-induced CNS injury. HS in rat models was induced by a high-temperature environment and treated with MSCs via the tail vein. The results demonstrated that MSC injection significantly reduced the mortality and inhibited the circulation inflammatory response. Moreover, the HS-induced neurological deficit and neuronic damage of the hippocampus were significantly ameliorated by MSC administration. In addition, MSC administration significantly restored astrocytes and inhibited cerebral inflammatory response. These results indicate that MSC infusion has therapeutic effects in HS of rats by regulating the circulation and cerebral inflammatory response. Moreover, astrocytes increased in MSC-treated HS rats when compared with the untreated ones. This may suggest a potential mechanism for HS prevention and therapy through MSC administration.

## Introduction

Heat stroke (HS) is the most serious type of heat illness which refers to the presence of hyperthermia (core body temperature that rises above 40°C) and central nervous system (CNS) dysfunction (1, 2). The morbidity of HS increases yearly and its mortality is over 60% (3, 4). In the early stages of HS, all patients have CNS injury, while more than 30% will then progress to long-term CNS dysfunction as sequela (1, 3, 5). The cerebellum and hippocampus of the brain are particularly vulnerable to HS which would induce a high rate of mortality or permanent neurological sequelae. Moreover, previous studies have identified that HS is a form of hyperthermia associated with a systemic inflammatory response that results in a syndrome of multi-organ dysfunction, coagulation activation, and fibrin formation which clinically manifests as disseminated intravascular coagulation (DIC) (1, 6, 7). In the CNS, HS induces hypotension and intracranial hypertension which reduces blood flow to the brain and results in cerebral ischemia, hypoxia, and neuronal damage. This neurotoxic cascade involves an overproduction of reactive oxygen species (ROS), cytokines, glutamate, and calcium ion (7, 8). Another study indicated that HS can decrease learning ability and memory in rats, the mechanisms of which may be related to changes of iron levels in the hippocampus (9). Despite pathogenesis and treatment having been deeply studied, including rapid, and effective cooling followed by close monitoring, blood purification therapy for renal/hepatic failure, continuous electroencephalogram (cEEG) monitoring, and the use of anticoagulants, unfortunately, no effective and comprehensive management for HS-induced organ dysfunction, especially for the CNS, has been developed (3, 10).

Currently, mesenchymal stem cell (MSC)-based therapy is being considered as a potential treatment strategy for CNS injury (11–13). MSCs possess multipotent properties, such as the capacity to secrete various factors, differentiation potential, and immunoregulatory effects (14). Previously, numerous studies on CNS injuries in animal models have shown that infused MSCs can ameliorate brain injuries (11–13). MSCs can protect against cerebral ischemic injury by preventing neuronal damage via the inhibition of apoptosis (15). Other animal experiments have demonstrated that MSCs play a potential neuronal protective effect in stroke, traumatic brain injury, multiple sclerosis, nerve injury, and neurodegenerative diseases via the secretion of neurotrophic factors, regulation of inflammatory reaction, modulation of ion metabolism, and so on (12, 13, 16, 17). These properties of MSCs make them excellent candidates for CNS injury management. Although previous studies have shown that mesenchymal stem cells could improve survival following HS in animals (12, 18, 19), the protective effects of brain injuries after HS has not been clearly demonstrated.

Therefore, in order to validate the effects of MSCs on brain injuries during HS, the present experiment was performed to assess the effects on survival, metabolic functions, neurons and astrocytes, and the plasma and brain levels of cytokines in rats with or without MSC administration after HS injury.

## Materials and Methods

### Animals

A total of 90 adult (aged 8 weeks) and 20 immature (aged 4 weeks) male Sprague-Dawley (SD) rats were supplied by the Experimental Animal Center of the General Hospital of PLA. All animal experiments were approved by the Animal Care and Use Committee of the General Hospital of PLA and were conducted in accordance with the NIH Guide for the Care and Use of Laboratory Animals. Animals were maintained in a room with a 12/12-h light/dark cycle and an ambient temperature of 22–25°C. All animals had free access to food and water. At the end of the experiment, the rats were anesthetized with pentobarbital sodium intraperitoneally (60 mg/kg) and sacrificed by cervical dislocation.

### Adipose-Derived MSC Isolation, Culture, and Identification

Adipose-derived MSCs were isolated and purified from the immature rats as previously described (20). Briefly, the rats were anesthetized with pentobarbital sodium intraperitoneally (60 mg/kg) and sacrificed by cervical dislocation. The adipose tissue isolated from the groin was cut into pieces and digested using 0.05% trypsin and 0.1% collagen I. The digest solution was filtered and centrifuged. After that, cells were washed twice and cultured in a low glucose DMEM (Gibco, Gran Island, NY, USA) supplemented with 10% fetal bovine serum (Gibco, Gran Island, NY, USA), penicillin (80 units/ml), and streptomycin (0.2 mg/ml). MSCs were identified as previously described (20). Immunologic phenotypes of third passage (P3) MSCs were analyzed by flow cytometry. Once an ~80% confluence was reached, cells were collected and counted, then were randomly divided into six groups (one group per antibody), each containing 1 × 10^6^ cells. Cells were then washed with PBS and incubated in the dark for 15 min at room temperature with the following antibodies: Allophycocyanin-conjugated CD90 (1:20; cat. no. 561409; BD Biosciences, San Jose, CA, USA), R-phycoerythrin-conjugated CD54 (1:20; cat. no. 554970; BD Biosciences), fluorescein isothiocyanate (FITC)-conjugated CD44 (1:20; cat. no. 550974; BD Biosciences), FITC-conjugated CD34 (1:20; cat. no. 11-0341-82; eBioscience; Thermo Fisher Scientific, Inc.), FITC-conjugated CD11b (1:20; cat. no. 554982; BD Biosciences), and FITC-conjugated CD45 (1:20; cat. no. 554877; BD Biosciences). Following incubation, cells were washed with PBS and then analyzed by flow cytometry, which was performed using a BD Accuri C6 software system (version 1.0.264.21; BD Biosciences).

In order to perform differentiation potential analysis, P3 MSCs were cultured in a six-well plate at a density of 10^4^ cells/well at 37 C in an atmosphere of 5% CO_2_ and relative humidity of ~100%. Once a ~70 or ~100% confluency for osteogenic or adipogenic differentiation was reached respectively, the medium was replaced with SD rat MSCs adipogenic (cat. no. RASMD-90031) or osteogenic (cat. no. RASMD-90021) differentiation medium (Cyagen Biosciences Inc., Guangzhou, China). Then at 37 C in an atmosphere of 5% CO2 and relative humidity of ~100%, the cells were cultured for 2 weeks. Following a fixation using 4% paraformaldehyde at room temperature for 30 min, adipogenic differentiation was detected by staining with 0.5% Oil red O (Sigma-Aldrich; Merck KGaA, Darmstadt, Germany) at room temperature for 1 h, and osteogenic differentiation was detected via staining with 0.1% alizarin red S (pH 4.2; Sigma-Aldrich; Merck KGaA) at room temperature for 30 min. An inverted microscope was used to observe the cells. The identification results of the cells are displayed in Supplementary Figure 1. The P2 MSCs cells were cryopreserved, and before intravenous infusion, they were resuscitated and then recultured into P4. Freshly harvested, the early passage (P4) MSCs were used in all subsequent experiments.

### HS Injury Rat Models Induction and Treatment

**Figure 1:**
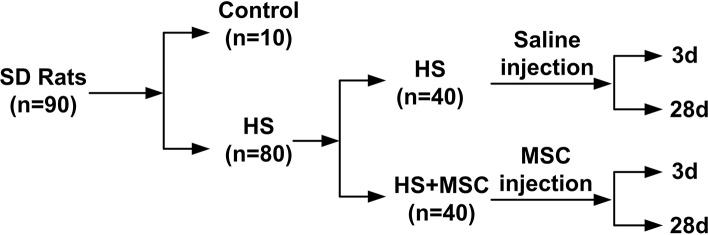
Study design.

A HS injury model was established as previously described (21). Briefly, the rats (without anesthesia) were maintained in a 40°C and 80% humidity environment. The rectal temperature of rat was monitored continuously. When temperatures reached 42°C (about 60 min), the rat was returned to room temperature and an intraperitoneal injection of 5 ml physiological saline was administered. Rats with paralysis and unconsciousness were identified as qualified HS injury models. The verified HS injury model rats were randomly divided into an HS group and an MSC-treated group (*n* = 40 in each group). Each rat in the treated group was infused with 2 × 10^6^ MSCs suspended in 0.3 ml physiological saline via the tail vein. The rats in the HS or MSC-treated groups were further randomly divided into early stage (3d) and late stage (28d) (*n* = 20 in each group). Besides, before HS injury models were built, 10 rats were randomly selected as control group and they were maintained at room temperature and only infused with 0.3 ml physiological saline (Figure 1). The rats were observed for 28 days after HS injury, with or without MSC infusion, to estimate the survival rates.

### Neurological Deficit Evaluation

Each rat in the different groups was evaluated for neurological deficits according to a modified Neurological Severity Score system containing motor (limb shape and walking posture), sensory (visual, tactile, and proprioceptive), reflex, and balance tests (22). Neurological function was graded on a scale of 0–10, and higher scores represent heavier injuries. Neurological Severity Score was assessed at the designated time point (i.e., 3 and 28d).

### Determination of the Effects of Infused MSCs on Cytokines in HS Injury Rats

For determination of interleukin-1β (IL-1β), interleukin-6 (IL-6), interleukin-10 (IL-10), tumor necrosis factor α (TNF-α), monocyte chemoattractant protein 1 (MCP-1), and growth-related oncogene (GRO), Rantes in the blood or brain tissues were taken at 3 days (early phase) and 28 days (late phase) after MSC infusion. Blood samples were centrifuged at 2000 g, for 10 min, at 4°C, and the supernatants were harvested. The brain samples were homogenized in 10 volumes of ice-cold PBS. The homogenates were centrifuged at 12,000 g, for 15 min, at 4°C. The supernatants were stored at −80°C until measurement. The concentration of IL-1β, IL-6, IL-10, TNF-α, MCP-1, and GRO, Rantes in the blood and tissue lysates were determined using Procarta Plex™ Analyst 1.0 (eBioscience, San Diego, USA) according to the manufacturers instruction.

### Histological Examinations

Perfusion fixation was performed to prepare brain tissue specimens at different time points after MSC infusion. Briefly, the rats were anesthetized and prepared with PBS or 4% paraformaldehyde. The brain tissues were dissected and immersed in 4% paraformaldehyde for 12 h and then immersed in a 30% of sucrose solution for 24 h at room temperature. The brain tissues were then frozen in a cooled embedding medium (OCT, Sakura Finetek USA, Inc.) and sliced into 7 μm-thick sections using a freezing microtome (Leica Microsystems, Wetzlar, Germany) for staining. The sections were stained with hematoxylin and eosin (HE) or Nissl's staining. These stained sections were visualized and scanned with a Panoramic MIDI CaseViewer System (3DHISTECH, Hungary).

### Immunofluorescence Staining

For immunofluorescence staining, polyclonal anti-glial fibrillary acidic protein (anti-GFAP) (1:100, Santa Cruz, CA, USA) was used as the primary antibody to identify GFAP expression or astrocytes. Briefly, sections were fixed in a cooled acetone for 15 min and washed with PBS twice. The sections were incubated with a 1 × blocking buffer (Cell Signaling Technology, Inc., Danvers, MA, USA) at 37°C for 1 h, and then incubated with a primary antibody overnight at 4°C, followed by further incubation for 1 h at 37°C with IgG Fab2 Alexa Fluor (R) 555 immuno-conjugated secondary antibody at a 1:1000 dilution (Cell Signaling Technology, Danvers, MA, USA). The nuclei were stained with 4′, 6-diamidino-2-phenylindole (DAPI, Vector Laboratories, Burlingame, CA, USA) for 15 min at room temperature. Negative controls were processed simultaneously by replacing the antibodies with PBS. The sections were visualized and scanned with a Panoramic MIDI CaseViewer System (3DHISTECH, Hungary).

### Statistical Analysis

SPSS 19.0 software was used to process the data. All experiments (biological replicates) were performed at least three times independently. Data were expressed as mean ± standard deviation. One-way ANOVA with Tukey's *post-hoc* analysis was used when data passed the test for normality and equal variance. If not, non-parametric statistical analysis (Wilcoxon signed-rank test) was used. Data for colonic temperature variables were analyzed using a repeated measures analysis of variance (time × group). A *p*-value of < 0.05 was considered to indicate a statistically significant difference.

## Results

### MSC Infusion Increased the Survival Rate of HS Rats

**Figure 2:**
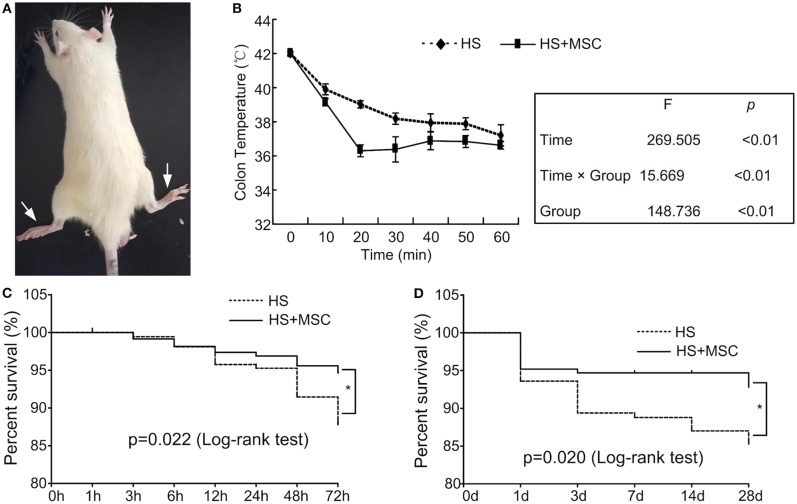
MSC infusion ameliorated the survival rate of HS rats. When the rectal temperature reached 42°C, the rat was returned to room temperature and an intraperitoneal injection of 5 ml physiological saline was administered. The HS rats were observed and validated to have acute hindlimb paralysis **(A)**. After MSCs administration, the rectal temperature of all HS rats was monitored continuously **(B)**. Moreover, the survival rate in the early (3 days) **(C)** and long-lasting stages (28 days) **(D)** was assessed. *n* = 40 in each group. *P < 0.05 vs. HS group.

The HS rats were observed and validated to have acute hindlimb paralysis (Figure 2A). Following MSC administration in the HS rats, colonic temperature decreased quickly when compared with the HS group rats (Figure 2B). Moreover, in the different stage phases, untreated HS rats demonstrated a higher mortality, whereas following the infusion of MSCs, the survival rate demonstrated a significant amelioration both in the early (3 days, Figure 2C) and long-lasting stages (28 days, Figure 2D). These results indicate that HS injury induced a paralysis symptom and high mortality in the rats, which could be ameliorated by MSC infusion.

### MSC Administration Modulated Pro-inflammatory and Anti-inflammatory Reactions of HS Rats

**Figure 3:**
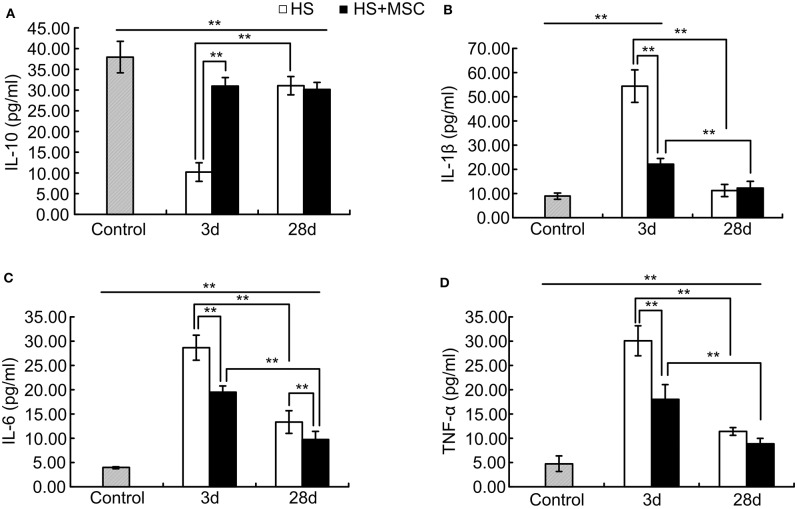
MSCs modulated pro-inflammatory and anti-inflammatory reactions of HS rats. At different time points after MSCs or saline infusion, the blood of rats in each group was collected, and the IL-10 **(A)**, IL-1β **(B)**, IL-6 **(C)**, and TNF-α **(D)** levels in the rat's blood serum were assayed. *n* = 10 rats per group. **P < 0.01.

As shown in Figure 3, following HS injury, the serum IL-10 levels were significantly decreased but IL-1β, IL-6, and TNF-α levels were significantly increased at 3 days as compared with the control rats (*P* < 0.01). This lasted until 28 days, where the IL-10 levels were still notably lower than that of control group (*P* < 0.01). However, they were significantly restored compared with the rats of the 3-day group (*P* < 0.01, Figure 3A). The IL-1β levels were approaching those of the control group and significantly decreased at 28 days when compared with the rats of 3-day group (*P* < 0.01, Figure 3B). The IL-6 and TNF-α levels were still notably higher than those of the control group (*P* < 0.01), but both significantly decreased compared with rats of the 3-day group (*P* < 0.01, Figures 3C,D). MSC-treated HS rats demonstrated a significant improvement in their levels of serum inflammatory cytokine. Compared with untreated rats, the serum IL-10 levels of the MSC-treated rats were significantly increased at 3 days and had no significant differences at 28 days (Figure 3A). The IL-1β, IL-6, and TNF-α levels of the MSC-treated rats were all significantly decreased when compared with those of the untreated rats at 3 days but showed no differences at 28 days except in IL-6 (Figures 3B–D). The IL-6 levels of the MSC-treated rats were still significantly decreased when compared with those of the untreated rats at 28 days (Figure 3C). These data demonstrate that MSC administration can modulate pro-inflammatory and anti-inflammatory reactions of HS rats.

### MSC Administration Provided Neuroprotective Effects for HS Rats

**Figure 4:**
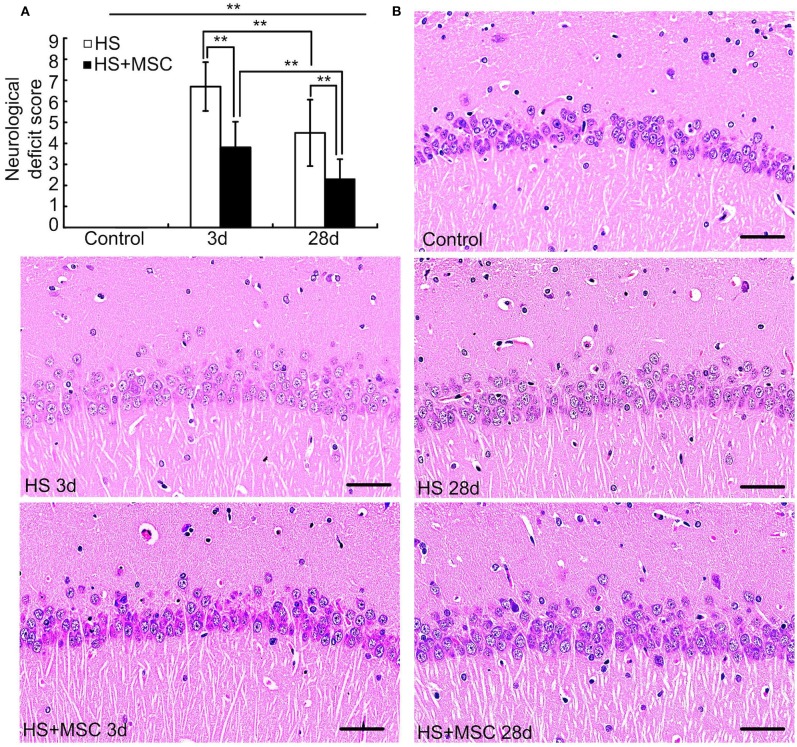
MSC administration provided neuroprotective effects for HS rats. At different time points after MSCs or saline infusion, the neurological deficit scores of rats in each group were tested **(A)**, *n* = 10 in each group, and the hippocampal histopathological properties were observed, *n* = 5 in each group, the representative images were shown, Scale bar, 50 μm **(B)**. **P < 0.01.

HS rats exhibited significantly increased neurological deficit scores at both 3 days and 28 days when compared with those of the control rats (both *P* < 0.01) (Figure 4A). However, the scores were significantly decreased at 28 days as compared with rats of the 3-day group (*P* < 0.01). MSC-treated HS rats also exhibited increased neurological deficit scores at both 3 and 28 days when compared with the control rats (*P* < 0.01), and the scores were notably decreased when compared with the same time point for untreated HS rats (both *P* < 0.01). Moreover, the hippocampal histopathological properties demonstrated that HS injury-induced neurons to perform a notably disorderly arrangement, loosened cytoplasms and karyopyknosis alterations, and the diffuse vacuolization, interstitial edema was also observed at both 3 and 28 days. The histopathological damage was especially more severe at 3 days than 28 days. Despite the MSC-treated HS rats also showing similar hippocampal histopathological damage, a significant amelioration was observed at both 3 and 28 days when compared with the same time points for untreated HS rats (Figure 4B).

### Astrocytes Increased in MSC-Treated HS Rats When Compared With the Untreated Ones

**Figure 5:**
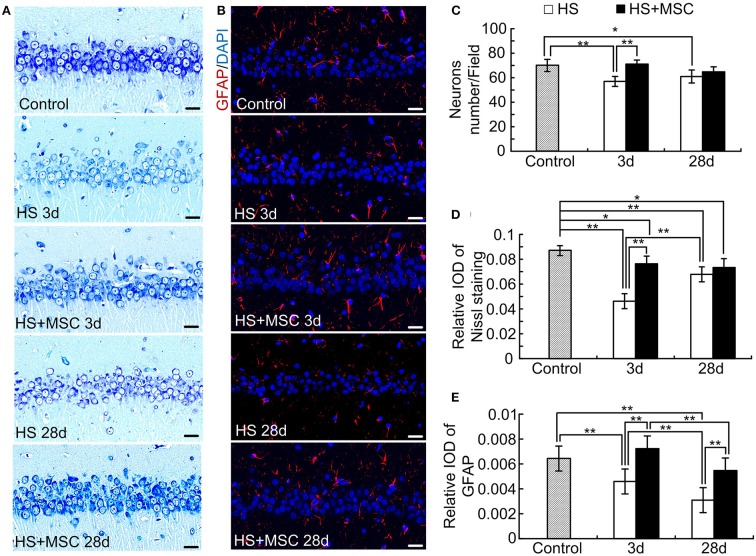
MSCs treatment ameliorated neurons and astrocytes in HS rats. At different time points after MSCs or saline infusion, the Nissl staining method in the hippocampus was used to assess the neuron alterations **(A)**, and the immunofluorescence staining of GFAP in the hippocampus was used to assess the astrocytes alterations **(B)**. The representative images were shown, Scale bar, 20 μm. The neuron count **(C)** and optical density of Nissl staining **(D)** and GFAP **(E)** were analyzed. *n* = 5 in each group, *P < 0.05, **P < 0.01.

As shown in Figure 5A, the cytoplasm of the control rats was trachychromatic and closely packed, the intracytoplasmic Nissl bodies were rich. After HS injury, the neurons performed a notably loosened arrangement and diffuse vacuolization, which was characterized by decreased density and an irregular arrangement of the nucleus and Nissl bodies at both 3 and 28 days. Especially, the diffuse vacuolization and decreased Nissl bodies were more notable in the brain of HS rats at 3 days than at 28 days. Despite the MSC-treated HS rats also showing similar properties of hippocampal neurons in Nissl staining, a significant amelioration was observed at both 3 and 28 days when compared with the same time point for untreated HS rats (Figures 5A,C,D). We further evaluated the astrocyte alterations in the brain of HS rats with or without MSC treatment through immunofluorescence staining of GFAP, which is an astrocyte specific marker. Accompanied by the neuronal alterations, notable decreases in GFAP expression, which indicates astrocytes loss, were observed at both 3 and 28 days after HS injury. MSC-treated HS rats showed approaching normal levels of GFAP expression at both 3 and 28 days when compared with the same time point for untreated HS rats (Figures 5B,E). These results suggested that astrocytes restored in the MSC-treated HS rats when compared with the untreated ones, which indicated the correlation between MSC treatment and astrocytes increment. The further relations and effects between them should be investigated deeply.

### MSC Administration Modulates Inflammatory and Chemotactic Cytokines in the Brain Tissue of HS Rats

**Figure 6:**
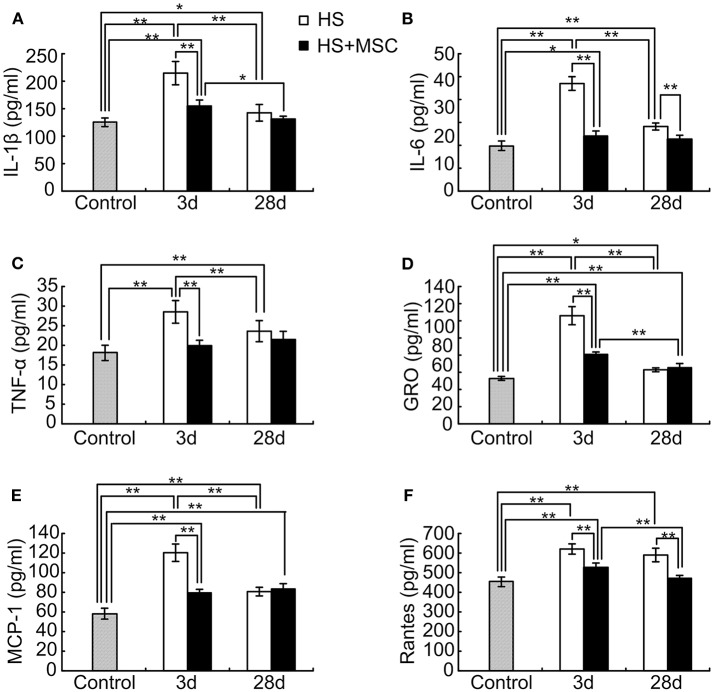
MSC administration modulates inflammatory and chemotactic cytokines in the brain tissue of HS rats. At different time points after MSCs or saline infusion, the brain tissue of rats in each group were collected and homogenized in 10 volumes of ice-cold PBS. The IL-1β **(A)**, IL-6 **(B)**, TNF-α **(C)**, IL-10 **(D)**, MCP-1 **(E)**, and Rantes **(F)** levels in the rat's brain tissue lysates were assayed. *n* = 10 rats per group. *P < 0.05, **P < 0.01.

Astrocytes are involved in the spatial buffering of many cytokines, signaling molecules, and energy sources that are implicated in the maintenance of homeostasis in the extracellular milieu of neurons. As neuronal damage was mitigated, accompanied with an increment of astrocytes, through MSC administration in HS rats, we further detected the inflammatory and chemotactic cytokines in the brain tissue of HS rats with or without MSC administration. As shown in Figure 6, following HS injury the levels of IL-1β, IL-6, and TNF-α in the rats' brains were significantly increased at 3 days when compared with those of the control rats (*P* < 0.01). Despite the IL-1β, IL-6, and TNF-α levels being notably decreased at 28 days when compared with those at 3 days (*P* < 0.01), they were still significantly increased when compared with that of the control group (*P* < 0.05 or 0.01). MSC-treated HS rats demonstrated a significant improvement in the levels of brain inflammatory cytokines. Compared with untreated rats, the levels of IL-1β and TNF-α in the brain tissue of MSC-treated rats were both significantly decreased at 3 days (*P* < 0.01), but had no differences at 28 days (Figures 6A,C). IL-6 levels of the MSC-treated rats were significantly decreased when compared with those of untreated rats at both 3 and 28 days (Figure 6B, *P* < 0.01). This lasted until day 28, when the above-mentioned cytokines all approached normal levels in the brains of MSC-treated HS rats (Figures 6A–C).

After HS injury, the rat's brain GRO, MCP-1, and Rantes levels were significantly increased at 3 and 28 days when compared with those of the control rats (*P* < 0.05 or 0.01). At 28 days, the GRO and MCP-1 both significantly decreased (*P* < 0.01), but Rantes had no differences when compared with rats of the 3-day group (Figures 6D–F). Compared with untreated rats, the brain GRO and MCP-1 levels of MSC-treated rats were both significantly decreased at 3 days but had no differences at 28 days (Figures 6D,E). The Rantes levels of MSC-treated rats were still significantly decreased when compared with those of the untreated rats at 28 days (Figure 6F). However, brain tissue GRO and MCP-1 levels were still notably higher, and Rantes levels had no differences when compared with those of the control group (Figures 6D–F).

## Discussion

Heat stroke (HS) is the most serious form of heat illness and is characterized by systemic organ damage and central nervous system (CNS) dysfunction (1, 2). The morbidity and mortality of HS are both increased yearly (3, 4). Unfortunately, no effective and comprehensive management for HS-induced organ dysfunction, especially for CNS dysfunction, exists. Our present study demonstrated that adipose-derived mesenchymal stem cell (MSCs) administration is a potential treatment strategy for HS-induced system inflammation and CNS injury in rats. Moreover, astrocytes increased in MSC-treated HS rats when compared with the untreated ones.

According to clinical data, the mortality of HS is over 60% (3, 4). Our present study indicated that HS rats were categorized by a high colonic temperature (42°C) and validated with acute hindlimb paralysis. Moreover, untreated HS rats exhibited a high mortality both in the early (3 days) and long-lasting stages (28 days). Following an infusion of MSCs, the colonic temperature of the rats decreased quickly; the survival rate demonstrated a significant amelioration both in the early (3 days) and long-lasting stages (28 days). These results indicate that HS injury induced a paralysis symptom and high mortality in rats, which could be ameliorated by MSC infusion. Weise et al. (23) reported a negative result when applying cryopreserved HUCB mononuclear cells into the treatment of stroke rats, which indicated potent problems in using cryopreserved cells. Actually, cryopreserved MSCs can be utilized in experiments in two ways. The cryopreserved cells can be resuscitated and then directly transfused into veins. Or the cells should be resuscitated and then recultured for several generations before intravenous infusion. The former way as Weise et al. (23) reported, might be prone to lead to negative results. In the present study, the latter way was selected, and the positive results suggested its effectiveness. Following the HS injury models induction, the decrease of the rats' temperature was related to inflammation modulation and physical heat dissipation. The phenomenon that the temperature of the treated group dropped more quickly than that of the HS group was observed, while the mechanism of it is still unclear and just based on conjecture. The infused MSC's prompt effect of modulating the system inflammatory reaction may contribute to the temperature decrease. But the cells should not have direct affection on the heat dissipation as they were very few in number and volume. The deeper mechanism on how infused MSC can cool down the HS rats so quickly is to be studied further. A previous study has also demonstrated that the survival rate of rats in the interstitial stem cell intervention group was significantly higher than in the HS group, and the earlier the mesenchymal stem cells were injected, the higher the survival rate (18). In addition, the survival rate, severity, and prognosis of HS were mainly associated with the intense system inflammatory reaction, increased vascular permeability, abnormal coagulation, cell dysfunction, and finally multi-organ failure (24–26). Therefore, MSC-mediated systemic inflammatory reactions in HS rats should be explored.

The results of the current study indicated that MSC infusion significantly restores serum IL-10 levels and inhibits the elevation of IL-1β, IL-6, and TNF-α levels at the early stage phase (3 days). At 28 days, the IL-6 levels were still significantly inhibited by the MSC infusion, but IL-10, IL-6, and TNF-α levels showed no significant differences between the MSCs-treated and untreated rats. High temperatures induced direct cell damage, secondary damage to the intestine and other organs, a coagulation disorder, and systemic inflammatory response syndrome which made the pathological process similar to sepsis (1, 6, 27). Sepsis is a potentially lethal syndrome that can induce both pro-inflammatory and anti-inflammatory mechanisms to become a disorder (28). In addition, previous studies have demonstrated that the cytokine-mediated systemic inflammatory response plays an important role in HS development (1, 6, 27). IL-1β, IL-6, and TNF-α are considered pro-inflammatory cytokines which can induce acute inflammatory reactions. Moreover, IL-6 and TNF-α levels were notably correlated with the severity and mortality in different animal HS models (29, 30). IL-10 is a key anti-inflammatory cytokine produced by activated immune cells, previous studies have demonstrated that treatment with CD34+ cells causes a significant increase in the serum levels of IL-10 during heatstroke (31). In addition, administration of human umbilical cord blood cells (HUCBC) also increases the serum levels of IL-10 and decreases the levels of TNF-α during heat stroke (19). These results demonstrate that the modulation of inflammatory cytokines in the serum may be an important role of MSCs against HS injury. MSCs possess multipotent properties, such as the capacity to secrete various factors, differentiation potential, and immunoregulatory effects (14). Many previous studies have confirmed that MSCs can perform notably anti-inflammatory effects, improving organ function, and ultimately reducing the mortality of sepsis (32–34). Our present study and other results raise the possibility that MSCs may be beneficial for protecting against multi-organ dysfunction during heat stroke.

CNS dysfunction or injury is a notable characteristic of HS. Our previous research has demonstrated that 100% of HS patients have CNS dysfunction, and the incidence of long-term neurological dysfunction reached 24.4% (35). Other research has also reported that all HS patients have CNS injury, while more than 30% of them will progress to long-term CNS dysfunction as sequela (3, 5). Our present study demonstrated that the neurological deficit and hippocampal histopathological properties were significantly ameliorated in MSC-treated HS rats at both 3 and 28 days when compared with the same time points for untreated HS rats. The neurological deficits were evaluated by a modified Neurological Severity Score system which is primarily used in ischemic stroke. However, Boltze et al. (36) reported that the system was not very sensitive and could be prone to compensation, which indicated the probable inaccuracy of the scores. Our present study showed that there were significant differences in scores between either 3d groups or 28d groups. The comparison between HS group and MSC group at the same time points (i.e., untreated 3d group vs. MSC-treated 3d group, untreated 28d group vs. MSC-treated 28d group) might offset partial compensation. Nevertheless, other behavior tests (such as the cylinder task, tapered beam walking, foot fault and Montoya's staircase) which are minimally affected by subjective scoring, compensation or repeated measurements should be considered to be applied to evaluate neurological deficits in further studies (37). The hippocampus of the brain is particularly vulnerable to various injury factors which would induce permanent neurological sequelae. During HS, heat damage can induce hypotension and intracranial hypertension which reduce blood flow to the brain and result in cerebral ischemia, hypoxia, and neuronal damage (19, 38). A secondary neurotoxic cascade which contains the reactive oxygen species (ROS), cytokines, glutamate, and calcium ion overload further exacerbates the neuronal damage (19, 38). Another researcher reported that HS can destroy learning ability and memory in rats, the mechanisms of which may be associated with iron level alterations in the hippocampus (9). Despite the pathogenesis being clear, rapid and effective treatments are still deficient. Currently, MSC-based therapy is being explored as a potential treatment strategy for CNS injury. MSCs can play potential neuronal protective effects in strokes, traumatic brain injuries, multiple sclerosis, nerve injuries, and neurodegenerative diseases (11–13). The specific mechanisms contain the secretion of neurotrophic factors, regulation of inflammatory reactions, modulation of ion metabolism, and so on (11–13). Therefore, the present results indicate that the neuroprotective effects by MSC infusion on HS rats may be associated with these MSC properties. In addition, we also demonstrated that MSC-treated HS rats exhibited a notable amelioration of hippocampal neurons, which was accompanied with restored astrocytes.

Astrocytes are involved in the spatial buffering of many ions, signaling molecules, and energy sources that are implicated in maintaining the homeostasis of the extracellular milieu of neurons. A previous study reported that murine astrocytes regulated chemokine expression and triggered cell survival *ex vivo* after HS (39). Another study found that MSC treatment restored astrocytic endfeet in the inflammatory brain (40). In our study, the increment of astrocytes in MSC-treated rats was founded. However, on the basis of present data, the mechanism of how MSCs reached the effects is still unclear. Besides, to validate whether astrocytes are responsible for the therapeutic effects, an astrocyte subtype analysis and more detailed investigation are required (41). Similar to other diseases, such as stroke, neuro-inflammation is the main contributor to CNS dysfunction in the HS condition. In the process of heat stress, pro-inflammatory factors and chemokines increase quickly, and a long-term increase in CNS cytokines and chemokines contribute to the consequences of CNS damage during heat stroke (42, 43). Our present study has demonstrated that MSC-treated HS rats demonstrated a significant improvement in the levels of brain inflammatory cytokines. Moreover, the brain GRO, MCP-1, and Rantes levels of MSC-treated rats were both significantly decreased. All of these results demonstrate that excessive activation of inflammation in the brain may be a major pathological mechanism of HS. Thereby, modulation of cerebral inflammatory response may be considered a potential management strategy for HS prevention and therapy.

In summary, the results of the present study indicate that MSC treatment may ameliorate the survival rate of rats by regulating circulation and cerebral inflammatory response. Moreover, astrocytes increased in MSC-treated HS rats when compared with the untreated ones. These may suggest a potential mechanism for HS prevention and therapy by MSC administration.

## Data Availability Statement

The datasets generated for this study are available on request to the corresponding author.

## Ethics Statement

The animal study was reviewed and approved by Animal Care and Use Committee of the General Hospital of PLA.

## Author Contributions

YuZ helped to design and perform the experiments and wrote the draft of the manuscript. ZD helped to design the study, perform the experiments, and analyze the results. YL helped to perform the experiments, analyze the results, and revise the manuscript. RY helped to perform the experiments. MY helped to analyze the results. YaZ helped to revise the manuscript. LW helped to perform the experiments. FZ helped to develop the study rationale and design. HK helped to conceive, design, and supervise the study and analyzed the results.

## Conflict of Interest

The authors declare that the research was conducted in the absence of any commercial or financial relationships that could be construed as a potential conflict of interest.
