# Supplementary material for: Mesenchymal Stem Cells Provide Neuroprotection by Regulating Heat Stroke-Induced Brain Inflammation
Source: Front Neurol. 2020 May 5;11:372. doi: 10.3389/fneur.2020.00372 (PMC7232542; doi:10.3389/fneur.2020.00372)
Supplement: Supplementary file 1 [file Table_1.DOCX]

Supplementary Material

## Supplementary Figure

**
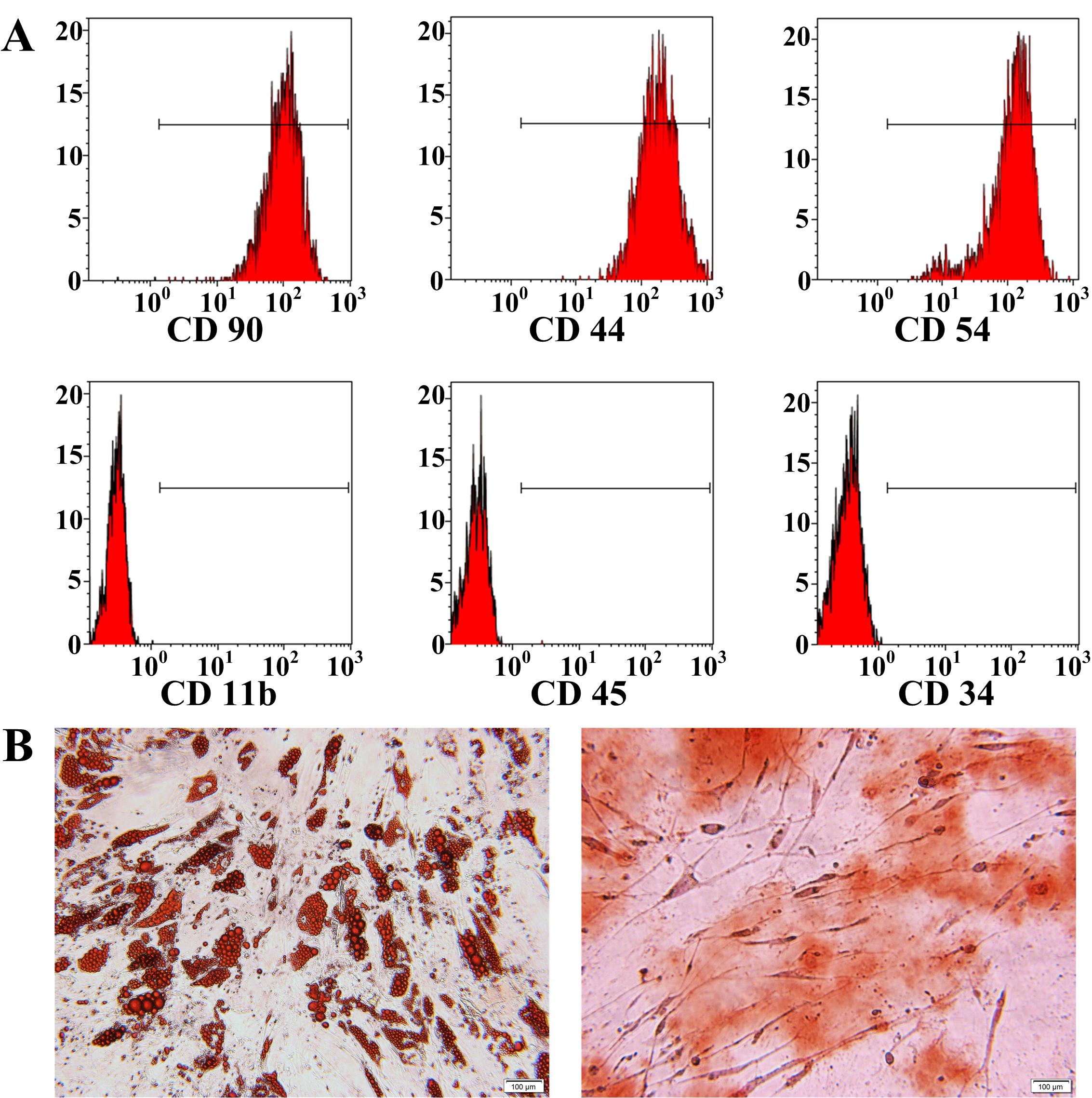
**

**Supplementary Figure 1.** Characteristics of adipose-derived MSCs. The MSCs were identified by their phenotypes and the potential for differentiating into adipocytes and osteoblasts. (A) For immunological phenotypes, the isolated and cultured cells were positive for CD90, CD44 and CD54, and negative for CD11b, CD45 and CD34. (B) Differentiation of cells into adipocytes and osteoblasts was confirmed by oil red O staining (the left image) and alizarin red staining (the right image), respectively. Scale bar, 100 μm. MSCs, mesenchymal stem cells.
